# 环状RNA UBAP2沉默对肺癌A549细胞体外增殖和侵袭的影响及机制

**DOI:** 10.3779/j.issn.1009-3419.2017.12.02

**Published:** 2017-12-20

**Authors:** 玉敬 殷, 辉 高, 佳 郭, 洋 高

**Affiliations:** 014030 包头，包头肿瘤医院病理科 Department of Pathology, Baotou Cancer Hospital, Baotou 014030, China

**Keywords:** circUBAP2, 肺肿瘤, A549细胞, 侵袭, circUBAP2, Lung neoplasms, A549 cells, Invasion

## Abstract

**背景与目的:**

已有的研究证明环状RNA（circular RNAs, circRNAs）在多种肿瘤发生发展中起重要作用，circUBAP2是一种促癌的circRNA，但是其在肺癌中的作用和机制尚不明确。本研究旨在探讨circular RNA UBAP2（circUBAP2）对人肺癌A549细胞体外增殖和侵袭能力的影响并初步探讨其机制。

**方法:**

应用CCK-8法检测circUBAP2表达沉默对A549细胞生长的抑制作用，采用流式细胞仪技术检测circUBAP2表达沉默对A549细胞周期和细胞失巢凋亡的影响，Transwell实验检测circUBAP2表达沉默对A549细胞侵袭能力的影响，Western blot和Real-time PCR检测CDK6、cyclin D1、p27、c-IAP1、Bcl-2、Survivin、Bax、FAK、Rac1、MMP2的表达变化以及JNK和ERK1/2的活性，荧光素酶报告技术检测circUBAP2直接靶基因。

**结果:**

CCK-8实验结果显示circUBAP2表达沉默后，A549细胞株增殖能力受抑制；流式细胞结果显示，circUBAP2表达沉默后，细胞周期停滞在G_0_期/G_1_期，且细胞凋亡率显著增加；Transwell实验结果显示，细胞体外侵袭能力明显受到抑制。Western blot和Real-time PCR结果显示，circUBAP2表达沉默后，*CDK6*、*cyclin D1*、*c*-*IAP1*、*Bcl*-*2*、*Survivin*、*FAK*、*Rac1*、*MMP2*等基因的表达水平显著下降，而p27和Bax的表达显著上升，而且JNK、ERK1/2的活性也受到明显的抑制，circUBAP2直接靶基因为miR-339-5p、miR-96-3p和miR-135b-3p。

**结论:**

circUBAP2在肺癌的体外增殖和侵袭中发挥重要的作用，抑制其表达，有望成为肺癌分子靶向治疗的新靶点。

## Abstract (en)

**Background and objective:**

It has been proven that circular RNAs (circRNAs) play an important role on the process of many types cancer and circUBAP2 was a cancer-promoting circRNA, however, the role and mechanism in lung cancer was not clear. The aim of this study is to investigate the effects of circUBAP2 on cell proliferation and invasion of human lung cancer A549 cells.

**Methods:**

CCK-8 assay was employed to detect the effect of circUBAP2 sliencing on cell proliferation of A549 cells. Fow cytometry was applied to detect the impact of circUBAP2 sliencing on cell cycle and cell anoikis, and Transwell invasion assay was applied to determine cell invasion of A549 cells. We also employed Western blot and Real-time PCR to determine the expressions of CDK6, cyclin D1, p27 and c-IAP1, Bcl-2, Survivin, Bax, FAK, Rac1 and MMP2, and the activities of JNK and ERK1/2, luciferase report gene assay was used to detect the targets.

**Results:**

CCK-8 assay showed that the inhibition of cell proliferation in the circUBAP2-siRNA group compared to untreated group and siRNA control group. Results of cell cycle detected by flow cytometry showed that cell cycle arrestd at G_0_/G_1_ after circUBAP2 silencing, cell apoptosis rate increased also. We also found that after circUBAP2 silencing, cell invasion of A549 cells was significantly inhibited. Western blot and Real-time PCR results showed that expression of CDK6, cyclin D1, c-IAP1, Bcl-2, Survivin, FAK, Rac1 and MMP2 were down-regulated, and the expression of p27 and Bax were up-regulated. Moreover, the activities of JNK and ERK1/2 were inhibited because of circUBAP2 silencing, the target genes were miR-339-5p, miR-96-3p and miR-135b-3p.

**Conclusion:**

CircUBAP2 plays an important role in the proliferation and invasion of human lung cancer. Silencing of circUBAP2 might be a novel target for molecular targeted therapy of patients with lung cancer.

环状RNA（circular RNAs, circRNAs）广泛地分布于人体各种组织，并在组织重塑、血管生成、胚胎形成以及肿瘤发生发展中起重要作用，环状RNA（circRNA）是一类特殊的非编码RNA分子，也是RNA领域最新的研究热点^[1]^。与传统的线性RNA不同，circRNA分子呈封闭环状结构，不受RNA外切酶影响，表达更稳定，富含microRNA结合位点，可通过miRNA海绵的作用解除miRNA对其靶基因的抑制作用，升高靶基因的表达水平，这一作用机制被称为竞争性内源RNA机制，在疾病中发挥着重要的调控作用^[2, 3]^。

近年来，circRNAs在肿瘤的作用受国内外学者的关注被认为与肿瘤的发生发展密切相关，如circRNAs HIPK3可通过调控miR-379促进NCI-H1299和NCI-H2170体外增殖，circRNAs ITCH可通过Wnt/β-Catenin调控肺癌细胞体外增殖^[4, 5]^。

肺癌是常见的恶性肿瘤之一，严重威胁着人类的健康，肺腺癌具有侵袭性强、预后差、多伴有淋巴结转移及易复发等特点。我国是肺癌发病率和死亡率最高的国家之一，为了进一步阐明circUBAP2在肺腺癌发生发展的作用，本文研究circUBAP2在肺腺癌组织和正常肺组织中的表达差异，通过siRNA干扰circUBAP2表达研究其对肺癌A549细胞增殖、细胞周期和侵袭的影响，并初步分析其可能的分子机制。

## 1 材料与方法

### 1.1 材料

肺腺癌和相对应的30例正常肺组织均通过病理学证实，取自包头肿瘤医院，并经液氮保存备用。人肺癌A549细胞购自中国科学院典型培养物保藏委员会细胞库（上海）；RPMI-1640培养基、胎牛血清、胰蛋白酶液、TRIzol及转染试剂Lipofectamine 3000均购自Life Technology公司（美国）；CCK-8试剂购自江苏碧云天生物技术有限公司（中国）；报告基因质粒pGL3-basic购自Promega公司（美国）；circUBAP2 siRNA质粒和对照siRNA质粒均购自上海吉玛制药技术有限公司；RT-PCR反转录试剂盒购自大连宝生物公司（中国）；细胞周期和凋亡Annexin Ⅴ-FITC/PI试剂盒均购自BD公司（美国）；CDK6、cyclin D1、p27、Bcl-2、Survivin、Bax、FAK、Rac1、MMP2和β-actin抗体均购自美国Santa Cruze公司（美国）；c-IAP1和JNK、ERK1/2抗体均购自CST公司（美国）。

### 1.2 方法

#### 1.2.1 siRNA转染

A549细胞常规培养于含10%的的胎牛血清的RPMI-1640培养液置于37 ℃、5%CO_2_、饱和湿度的培养箱中。当细胞融合度达50%左右时，按照Lipofectamine 3000的说明书进行，将50 nmol/L circUBAP2 siRNA质粒和对照siRNA转染肺癌A549细胞，转染48 h后EDTA-胰酶消化，进行后续实验。实验时将细胞分为3组：未处理组（只加Lipofectamine 3000的A549细胞）、对照siRNA组（转染对照siRNA的A549细胞）、siRNA组（转染circUBAP2 siRNA质粒的A549细胞）。

#### 1.2.2 CCK-8方法检测细胞增殖

分别收集转染24 h、48 h和72 h后A549细胞铺于96孔板中，每个时点5个复孔，按CCK-8的操作说明书进行，加入CCK-8试剂于37 ℃中继续培养2 h，然后与酶标仪上测定450 nm的吸光度。

#### 1.2.3 流式细胞术检测细胞周期

A549细胞转染48 h后，用0.25%的胰酶消化，2, 000 r/min离心5 min收集细胞，PBS洗2次，2, 000 r/min离心5 min收集细胞，500 μL 70%乙醇固定，4 ℃过夜。第2天，将乙醇固定的细胞4 ℃ 2, 000 r/min离心5 min，弃上层乙醇溶液。采用冷的PBS洗细胞2次，每管加50 g/L的PI溶液500 μL重悬细胞沉淀，室温避光静置30 min染色，BD流式细胞仪检测细胞周期分布。

#### 1.2.4 流式细胞术检测细胞凋亡率

A549细胞转染48 h后，铺于用poly-HEMA包被的6孔板，培养48 h，离心收集，以预冷的PBS洗涤，按Annexin Ⅴ-FITC/PI细胞凋亡试剂盒说明书进行，BD流式细胞仪进行凋亡检测。

#### 1.2.5 Real-time PCR检测circUBAP2下游靶基因的变化

肺癌和相对应的正常肺组织总RNA，以及细胞总RNA均采用TRIzol试剂（Life Technology公司）进行提取，紫外分光光度仪测定其浓度。取总RNA 2 μg，按M-MLV逆转录试剂盒操作说明合成cDNA。以GAPDH作为内参照，ABI 7500荧光定量PCR仪上SYBR Green Ⅰ染料检测各基因的表达情况。反应程序：94 ℃变性3 min后，按下述条件扩增35个循环：95 ℃ 5 s，退火温度30 s，72 ℃ 95 s，72 ℃延伸5 min。采用2^-△△CT^法分析各个样品的基因相对表达差异，各基因引物序列如下表，引物由上海生工生物工程有限公司合成并纯化。

#### 1.2.6 Western blot检测circUBAP2下游靶蛋白的变化

收集转染后48 h后的A549细胞，加入细胞裂解液和蛋白酶抑制剂PMSF后离心收集总蛋白，经10%SDS-PAGE电泳后转移至PVDF膜上，4 ℃、5%脱脂牛奶封闭1 h后，加入CDK6（1:2, 000）、cyclin D1（1:2, 000）、p27（1:2, 000）、c-IAP1（1:2, 000）、Bcl-2（1:2, 000）、Survivin（1:2, 000）、Bax（1:2, 000）、FAK（1:2, 000）、Rac1（1:2, 000）、MMP2（1:2, 000）、p-JNK（1:800）、p-ERK1/2（1:600）、JNK（1:1, 500）、ERK1/2（1:1, 500）和β-actin（1:5, 000）抗体4 ℃孵育过夜。TBST洗去一抗，IgG-HRP标记二抗室温孵育1 h，TBST洗涤3次，ECL试剂盒显影，以β-actin作为内参。

#### 1.2.7 荧光素酶报告试验

本研究中已通过TargetScan软件预测circRNA UBAP2可能结合miR-339-5p、miR-1296-5p、miR-96-3p、miR-215-3和pmiR-135b-3p，降低上述miRNAs丰度，本研究将线性UBAP2序列5’-CTATCAATATATTGCTGGAAGGGAATTCAGACACAGCAACAGCTGAACAGATGCGTCTCG-3’构建至荧光素酶报告基因载体pGL3-basic，将circRNA UBAP2、UBAP2和上述miRNAs分别共转染至细胞，37 ℃、5%CO_2_条件下培养12 h，以酶标仪观测荧光强度的变化。

### 1.3 统计学分析

数据处理采用SPSS 11.0统计软件，计量资料用计量资料用均数±标准差（Mean±SD）表示，组间比较采用*t*检验或单因素重复测量设计的方差分析（*One*-*way ANOVA*），*P*＜0.05表示差异具有统计学意义。

## 2 结果

### 2.1 circUBAP2在肺癌组织中高表达

Real-time PCR结果显示，肺腺癌组织中circUBAP2的相对表达量与正常肺组织差异有统计学意义（*t*=4.743, *P*=0.001），肺腺癌组织中circUBAP2的相对表达量高于正常肺组织（图 1）。

**1:**
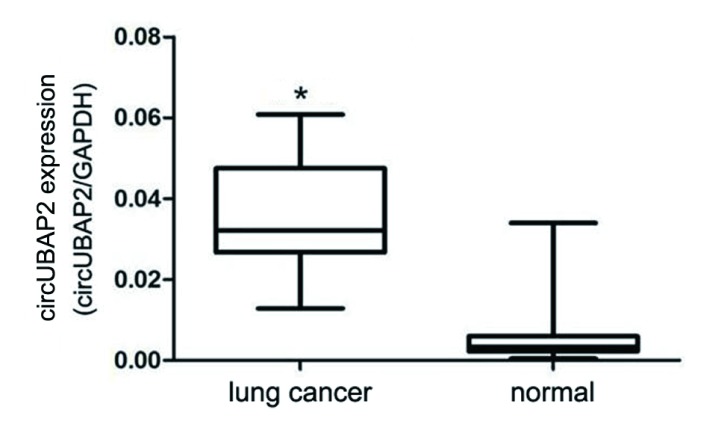
肺癌组织及其对应的正常肺组织中circUBAP2的表达水平。与正常组相比，^*^P=0.001。 The expression of circUBAP2 in human lung cancer and normal tissues. Compared with normal group, ^*^P=0.001.

### 2.2 circUBAP2沉默A549细胞株的建立

Real-time PCR检测结果显示（图 2），A549细胞circUBAP2 siRNA组中circUBAP2的表达水平与未处理组和对照组差异有统计学意义（*F*=37.901, *P*＜0.001）。

**2:**
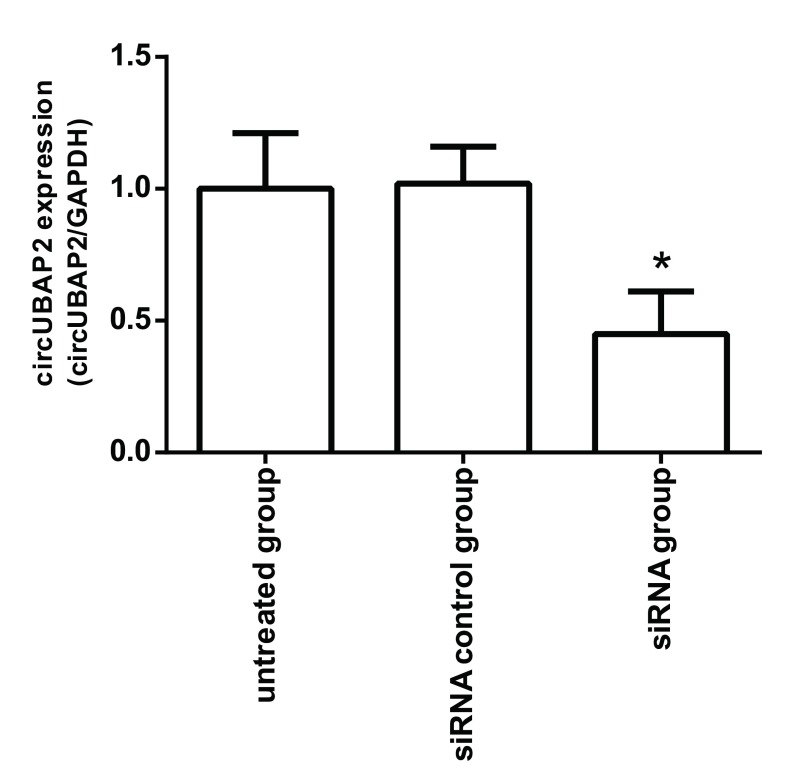
circUBAP2 siRNA对A549细胞中circUBAP2表达的影响。与未处理组相比，^*^P＜0.05。 The effect of circUBAP2 siRNA on circUBAP2 expression in A549 cells. Compared with untreated group, ^*^P < 0.05.

### 2.3 circUBAP2沉默对A549细胞增殖的影响

细胞增殖水平以OD值表示，实验组与未处理组在24 h、48 h、72 h OD值比较，采用重复测量数据的方差分析，结果：①不同时间点间的OD值有差别（*F*=33.581, *P*＜0.001），②实验组与未处理组OD值有差别（*F*=84.692, *P*＜0.001），实验组与未处理组相比OD值比较低，细胞增殖受到抑制。③实验组与未处理组的OD值变化趋势有差别（*F*=6.133, *P*=0.021），见图 3。

**3:**
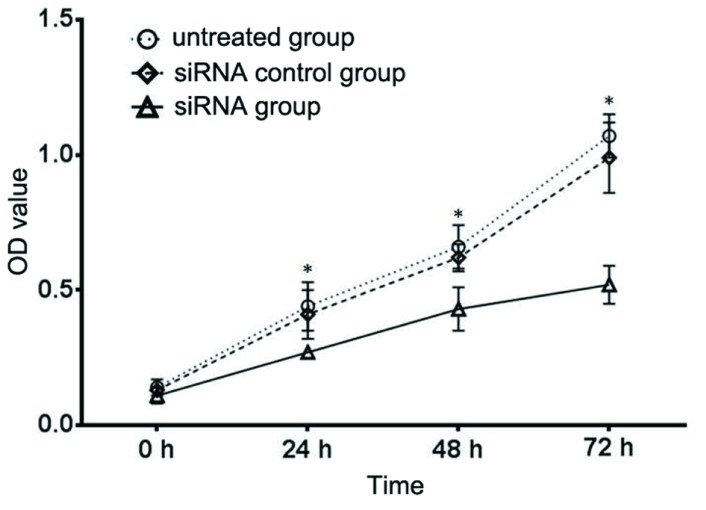
circUBAP2沉默对A549细胞增殖的影响。与未处理组相比，^*^P＜0.05。 The effect of circUBAP2 siRNA on cell proliferation in A549 cells. Compared with untreated group, ^*^P < 0.05.

**1:** Real-time PCR引物及退火温度 Real-time primers and anealing temperatures

Gene	Primers sequences	Anealing temperature (℃)
*circUBAP2*	F: 5’-AGCCTCAGAAGCCAACTCCTTTG-3’	60
	R: 5’-TCAGGTTGAGATTTGAAGTCAAGAT-3’	
*CDK6*	F: 5’-TCTTCATTCACACCGAGTAGTGC-3’	60
	R: 5’-TGAGGTTAGAGCCATCTGGAAA-3’	
*Cyclin D1*	F: 5’-GGCGGATTGGAAATGAACTT-3’ R: 5’-TCCTCTCCAAAATGCCAGAG-3’	58
*P27*	F:5’-CATTCCATGAAGTCAGCGAT-3’	58
	R: 5’-CGTCAAACGTAAACAGCTCG-3’	
*c*-*IAP1*	F: 5’-CATTCCATGAAGTCAGCGAT-3’	59
	R: 5’-TGCTTTTGTTGTGATGGTGG-3’	
*Bcl*-*2*	F: 5’-CAGCCAGGAGAAATCAAACAG-3’	60
	R: 5’-GACTGAGTACCTGAACCGGC-3’	
*Survivin*	F: 5’-TCCGCAGTTTCCTCAAATTC-3’	60
	R: 5’-GTTGCGCTTTCCTTTCTGTC-3’	
*Bax*	F: 5’-CGGCGAATTGGAGATGAACTG-3’	60
	R: 5’-AGCAAAGTAGAAGAGGGCAACC-3’	
*FAK*	F: 5’-GCTCCACCAAAGAAACCG-3’	56
	R: 5’-GCCCGTCACATTCTCGTAC-3’	
*Rac1*	F: 5’-TGTAGTCGCTTTGCCTATTGATG-3’	59
	R: 5’-CATCGTCAGCACTAGCACAGTTT-3’	
*MMP2*	F: 5’-TGATCTTGACCAGAATACCATCGA -3’	60
	R: 5’-GGCTTGCGAGGGAAGAAGTT-3’	
*GAPDH*	F: 5’-GGAGCGAGATCCCTCCAAAAT-3’	60
	R: 5’-GGCTGTTGTCATACTTCTCATGG-3’	

**2:** circUBAP2沉默对A549细胞mRNA表达的影响 The effect of circUBAP2 siRNA on mRNA expression in A549 cells

Gene	Untreated group	siRNA control group	siRNA group	*F*	*P*
*CDK6*	1.000±0.092	0.922±0.132	0.682±0.081	43.031	＜0.001
*Cyclin D1*	1.000±0.152	0.951±0.131	0.521±0.052	58.390	＜0.001
*c*-*IAP1*	1.000±0.083	0.881±0.142	0.642±0.052	47.272	＜0.001
*P27*	1.000±0.111	0.943±0.152	2.512±0.204	758.100	＜0.001
*Bcl*-*2*	1.000±0.122	1.112±0.082	0.462±0.051	70.490	＜0.001
*Survivin*	1.000±0.134	0.891±0.161	0.411±0.082	91.241	＜0.001
*Bax*	1.000±0.162	1.092±0.112	1.662±0.111	197.300	＜0.001
*FAK*	1.000±0.131	0.910±0.16	0.471±0.051	68.141	＜0.001
*Rac1*	1.000±0.112	1.141±0.16	0.490±0.071	62.142	＜0.001
*MMP2*	1.000±0.181	1.082±0.15	0.531±0.062	52.262	＜0.001

### 2.4 circUBAP2沉默对A549细胞周期分布的影响

流式细胞仪结果显示，circUBAP2 siRNA组中A549细胞在G_0_期/G_1_期比例为（71.21±5.29）%，而未处理组和对照siRNA组的比例分别为（49.07±5.83）%和（46.58±4.24）%，采用单因素重复测量设计的方差分析和t检验差异有统计学意义（*F*=22.802, *P*＜0.001），circUBAP2 siRNA组G_0_期/G_1_期比例高于未处理组（图 4）。

**4:**
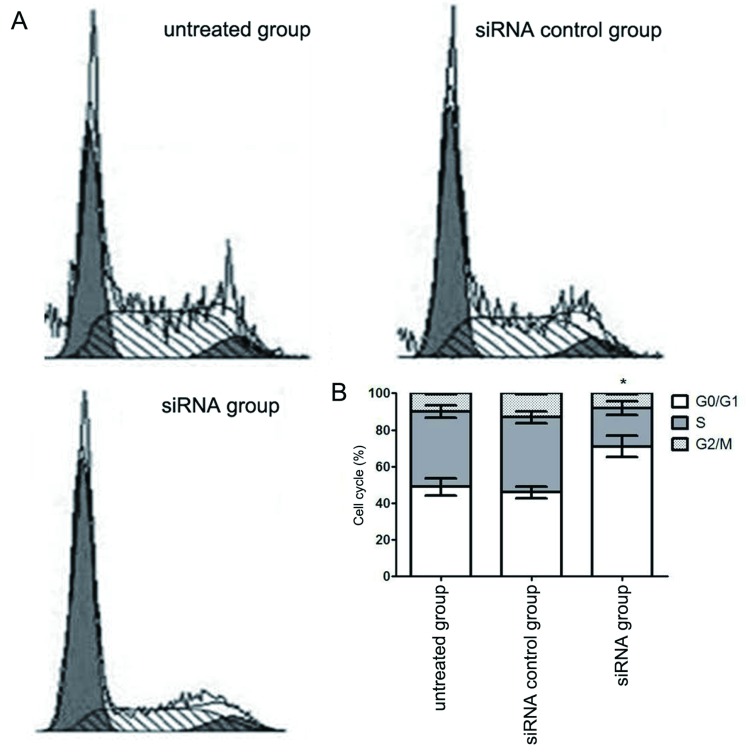
circUBAP2沉默对A549细胞周期的影响（*n*=3）。A: circUBAP2沉默后A549细胞流式细胞术周期检测结果；B：circUBAP2沉默后A549细胞G_0_期/G_1_期比例显著升高。与未处理组相比，^*^P＜0.05。 The effect of circUBAP2 siRNA on cell cycle in A549 cells (*n*=3). A: The flow cytometry results showed the cell cycle of circUBAP2 siRNA in A549 cells; B: The G_0_/G_1_ ratio was significantly increased after circUBAP2 siRNA in A549 cells. Compared with untreated group, ^*^P < 0.05.

### 2.5 circUBAP2沉默对A549细胞失巢凋亡的影响

流式细胞仪结果显示（图 5），circUBAP2 siRNA组中A549细胞的凋亡率为（49.11±3.62）%，与未处理组（23.74±2.62）%和与对照siRNA组（24.55±3.11）%相比差异有统计学意义（*F*=84.210, *P*＜0.001）。

**5:**
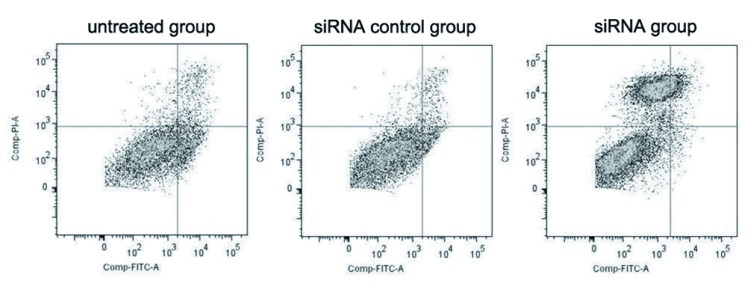
circUBAP2沉默对A549细胞失巢凋亡的影响 The effect of circUBAP2 siRNA on cell anoikis in A549 cells

### 2.6 circUBAP2沉默对A549细胞侵袭能力的影响

Transwell侵袭实验表明，circUBAP2 siRNA干扰后，其细胞侵袭力相对与对照组明显降低（图 6），circUBAP2 siRNA组中A549细胞的侵袭率为（38.72±4.11）%，与未处理组（100.0±10.21）%与对照siRNA组（97.60±8.62）%相比差异有统计学意义（*F*=92.460, *P*＜0.001），表明沉默circUBAP2可抑制肺癌A549细胞的体外侵袭能力。

**6:**
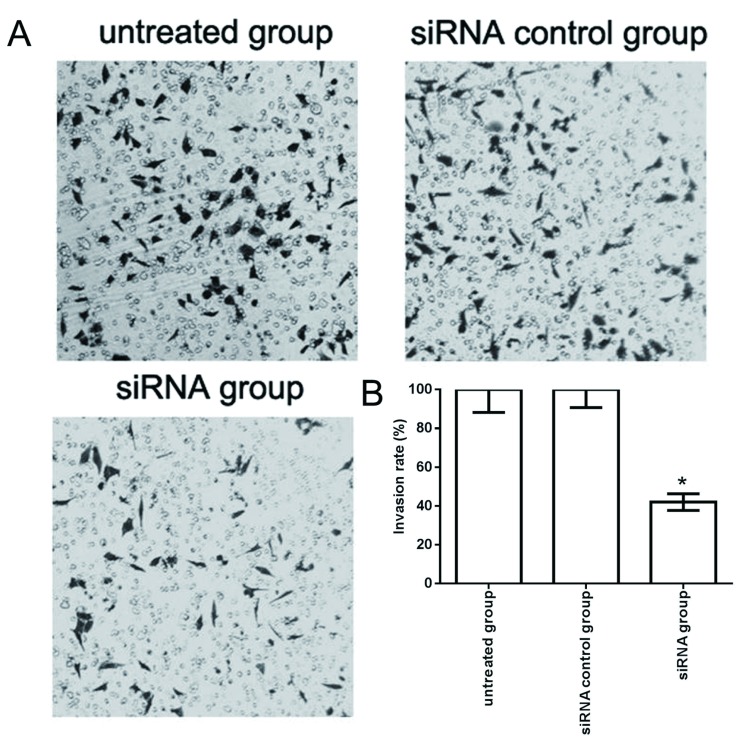
circUBAP2沉默对A549细胞侵袭能力的影响（×200）。A：circUBAP2沉默后A549细胞Transwell侵袭检测结果；B：circUBAP2沉默后A549细胞侵袭率显著下降。与未处理组相比，^*^P＜0.05。 The effect of circUBAP2 siRNA on invasion in A549 cells (×200). A: The Transwell results showed the invasion of circUBAP2 siRNA in A549 cells; B: The invasion rate was significantly decreased after circUBAP2 siRNA in A549 cells. Compared with untreated group, ^*^P < 0.05.

### 2.7 circUBAP2沉默处理对细胞周期相关基因表达的影响

Western blot检测和Real-time PCR结果显示，与未处理组细胞相比，circUBAP2 siRNA处理后A549细胞中的周期凋亡相关基因*CDK6*、*cyclin D1*、*c*-*IAP1*、*Bcl*-*2*和*Survivin*的mRNA和蛋白的表达水平显著下降，而p27和Bax的表达显著上升。干扰组中转移相关基因中*FAK*、*Rac1*和*MMP2*的表达较对照组都显著下降，采用单因素重复测量设计的方差分析和t检验差异有统计学意义（图 7）。

**7:**
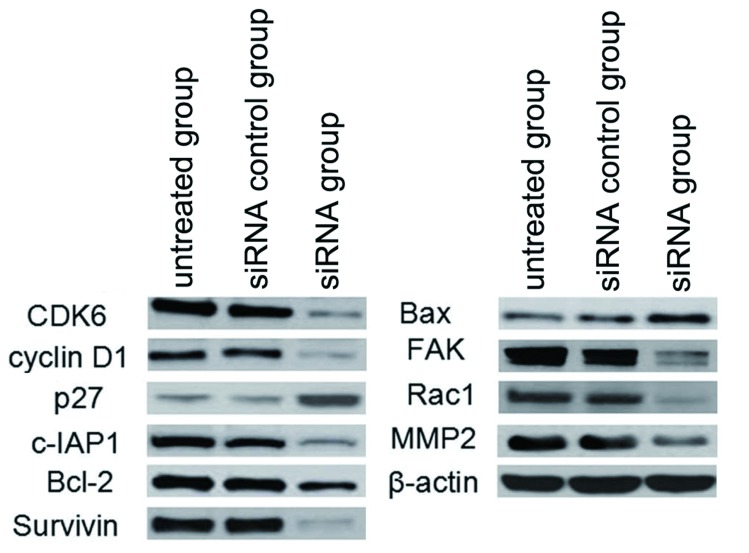
circUBAP2沉默对细胞凋亡、转移相关基因表达的影响 The effect of circUBAP2 siRNA on cell apoptosis and metastasis related genes in A549 cells

### 2.8 circUBAP2沉默对JNK-MAPK信号途径的影响

我们采用Western blot检测了ERK1/2和JNK的活性表达。结果表明，circUBAP2干扰可下调JNK和ERK1/2的活性（图 8），提示JNK-MAPK信号途径在circUBAP2调控肺癌细胞侵袭转移过程中起关键作用。

**8:**
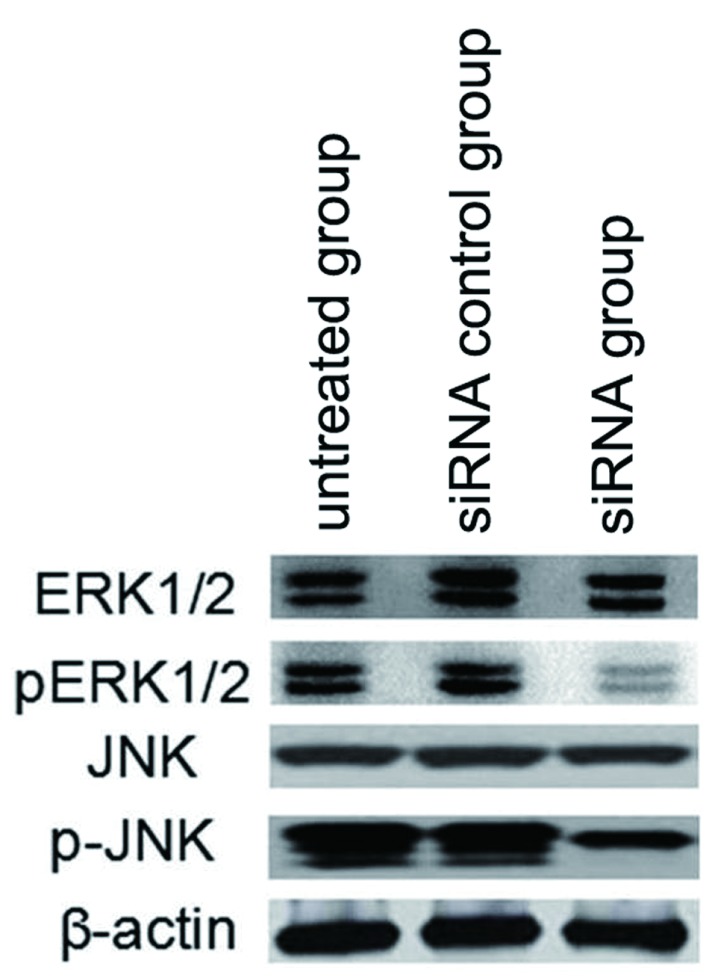
circUBAP2沉默对JNK-MAPK信号途径的影响 The effect of circUBAP2 siRNA on JNK-MAPK pathway in A549 cells

### 2.9 circUBAP2直接作用miRNA

我们采用荧光素酶实验分析了circUBAP2直接作用miRNA。结果表明，circUBAP2可挽救miR-339-5p、miR-96-3p和miR-135b-3p对UBAP2荧光的抑制作用（图 9），则表明circUBAP2可与上述miRNA相结合，miR-339-5p、miR-96-3p和miR-135b-3p是circUBAP2直接作用miRNA。

**9:**
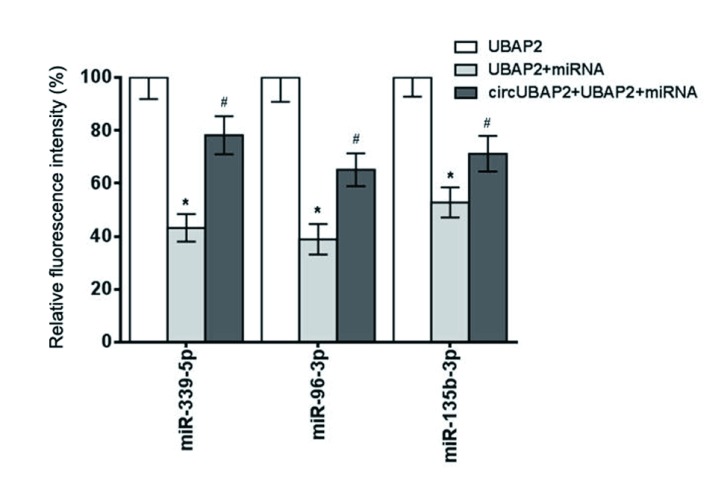
circUBAP2在A549细胞中的直接作用miRNA。与UBAP2组相比，^*^P＜0.05；与UBAP2+miRNA组相比，^#^*P*＜0.05。 The direct miRNA of circUBAP2 in A549 cells. Compared with UBAP2 group, ^*^P < 0.05; compared with UBAP2+miRNA group, ^#^*P* < 0.05.

## 3 讨论

研究显示，circUBAP2与肿瘤的发生发展有关，参与肿瘤的形成、侵袭和转移，其在肿瘤中可能扮演癌基因的作用^[6]^，但circUBAP2在肺癌的作用机制尚不清楚。肺腺癌是恶性程度较高的肺癌类型，因此，本研究首先检查了30例肺腺癌组织及对应的正常肺组织中circUBAP2的表达水平，发现circUBAP2在肺腺癌组织中的表达明显高于正常肺组织，提示circUBAP2在肺腺癌发生发展中发挥重要作用，但其确切的作用机制需要进一步探讨。

为了进一步研究circUBAP2在肺腺癌细胞中的作用，我们采用siRNA干扰技术沉默circUBAP2的表达，体外研究circUBAP2对肺癌A549细胞增殖、细胞周期、细胞失巢凋亡和细胞侵袭能力的影响，结果发现circUBAP2表达沉默能抑制肺癌细胞体外增殖及侵袭能力，并且诱导细胞周期G_0_期/G_1_期停滞和细胞失巢凋亡，进一步证实circUBAP2在肺癌细胞中扮演癌基因的作用。

为了进一步探讨circUBAP2表达下调介导的细胞生长和侵袭能力受抑制的可能分子机制，我们研究了细胞增殖和凋亡相关基因*CDK6*、*cyclin D1*、*p27*、*c*-*IAP1*、*Bcl*-*2*、*Survivin*和*Bax*表达情况，结果发现*CDK6*、*cyclin D1*和*c*-*IAP1*的表达显著下调，而*p27*和*Bax*显著上升。周期蛋白依赖性蛋白激酶CDK6是细胞周期调控中的重要因子，和cyclin D结合形成异二聚体，而实现对G_1_期-S期的推进和转化作用^[7]^。P27可通过结合cyclin-CDK2复合物和cyclin-CDK4复合物并抑制其活性，调控p53/p27信号通路参与肿瘤细胞细胞周期的调控将细胞周期阻滞在G_0_期/G_1_期^[8-11]^。

失巢凋亡（anoikis）是一种由细胞外基质和其他细胞脱离接触而诱发的细胞程序性死亡，在机体发育、组织自身平衡、疾病发生和肿瘤转移中起重要作用。因此考察circUBAP2对肺癌细胞失巢凋亡的影响，可以更加真实地反映其在体内调控肿瘤细胞凋亡的能力，本研究发现沉默circUBAP2的表达可诱导A549细胞失巢凋亡。c-IAP1是抗细胞凋亡因子家族的重要成员之一，可通过抑制Caspase3、7、9等酶的活性而发挥阻断凋亡的作用^[12]^。Bcl-2、Survivin和Bax是细胞凋亡的重要调控因子^[13]^，circUBAP2可能通过调控上述基因的表达参与肺癌细胞的失巢凋亡。

Rac1是细胞内重要的信号转导分子，通过GDP/GTP循环发挥其生物学功能，参与细胞运动、侵袭转移等细胞行为。*FAK*是Rac1的重要靶基因，是Rac-FAK1信号途径的关键基因，在细胞侵袭转移中发挥重要作用。MMP-2是主要的Ⅳ型基质金属蛋白酶，与肿瘤的生长、浸润及转移密切相关，也是Rac-FAK1信号途径的重要下游基因^[14]^。本研究中，circUBAP2干扰可抑制Rac1和FAK的表达，并进而抑制MMP-2的表达，提示circUBAP2可能通过Rac-FAK1信号通路调控肿瘤细胞的侵袭和转移。

JNK信号途径在肿瘤细胞侵袭中起着重要作用，其中JNK和ERK1/2为该途径的关键部位^[15]^。本研究显示，circUBAP2干扰可抑制JNK和ERK1/2的活性，提示JNK信号途径在circUBAP2调控肺癌细胞侵袭转移过程中起关键作用。环状RNA分子具有miRNA作用位点，可以通过miRNA海绵作用竞争性的与miRNA结合，从而间接调控miRNA靶基因的表达，被结合的miRNA可以被认为是环状RNA的直接作用miRNA。本研究通过荧光素酶实验分析了circUBAP2直接作用miRNA。结果表明miR-339-5p、miR-96-3p和miR-135b-3p是circUBAP2直接作用miRNA。

总之，circUBAP2在肺癌细胞中发挥重要作用，沉默circUBAP2可抑制肺癌细胞的体外生长、侵袭，并诱导细胞周期停滞和凋亡，其可能成为肺癌治疗的重要靶点。
